# High burden of malaria following scale-up of control interventions in Nchelenge District, Luapula Province, Zambia

**DOI:** 10.1186/1475-2875-13-153

**Published:** 2014-04-23

**Authors:** Victor M Mukonka, Emmanuel Chanda, Ubydul Haque, Mulakwa Kamuliwo, Gabriel Mushinge, Jackson Chileshe, Kennedy A Chibwe, Douglas E Norris, Modest Mulenga, Mike Chaponda, Mbanga Muleba, Gregory E Glass, William J Moss

**Affiliations:** 1 Department of Public Health, Copperbelt University, School of Medicine, Ndola, Zambia; 2 Ministry of Health, National Malaria Control Centre, Lusaka, Zambia; 3 W Harry Feinstone Department of Molecular Microbiology & Immunology, Johns Hopkins Bloomberg School of Public Health, Baltimore, USA; 4 Zambian Ministry of Environment and Statistics, Lusaka, Zambia; 5 District Health Office, Nchelenge, Zambia; 6 Luapula Provincial Medical Office, Mansa, Zambia; 7 Tropical Diseases Research Centre, Ndola, Zambia; 8 Department of Epidemiology, Bloomberg School of Public Health, Johns Hopkins University, Baltimore, Maryland, USA

## Abstract

**Background:**

Malaria control interventions have been scaled-up in Zambia in conjunction with a malaria surveillance system. Although substantial progress has been achieved in reducing morbidity and mortality, national and local information demonstrated marked heterogeneity in the impact of malaria control across the country. This study reports the high burden of malaria in Nchelenge District, Luapula Province, Zambia from 2006 to 2012 after seven years of control measures.

**Methods:**

Yearly aggregated information on cases of malaria, malaria deaths, use of malaria diagnostics, and malaria control interventions from 2006 to 2012 were obtained from the Nchelenge District Health Office. Trends in the number of malaria cases, methods of diagnosis, malaria positivity rate among pregnant women, and intervention coverage were analysed using descriptive statistics.

**Results:**

Malaria prevalence remained high, increasing from 38% in 2006 to 53% in 2012. Increasing numbers of cases of severe malaria were reported until 2010. Intense seasonal malaria transmission was observed with seasonal declines in the number of cases between April and August, although malaria transmission continued throughout the year. Clinical diagnosis without accompanying confirmation declined from 95% in 2006 to 35% in 2012. Intervention coverage with long-lasting insecticide-treated nets and indoor residual spraying increased from 2006 to 2012.

**Conclusions:**

Despite high coverage with vector control interventions, the burden of malaria in Nchelenge District, Zambia remained high. The high parasite prevalence could accurately reflect the true burden, perhaps in part as a consequence of population movement, or improved access to care and case reporting. Quality information at fine spatial scales will be critical for targeting effective interventions and measurement of progress.

## Background

Zambia is a malaria-endemic country in sub-Saharan Africa with an estimated 4.5 million malaria episodes and 7,737 malaria-related deaths in 2011 [1,2]. The majority of malaria episodes were caused by *Plasmodium falciparum*[1] and the major malaria vectors are *Anopheles gambiae, Anopheles arabiensis* and *Anopheles funestus*[3]. To reduce the disease burden, several malaria control interventions were scaled-up from 2006–2011, including case management with rapid diagnostic tests (RDTs) and artemisinin-combination therapy (ACT), distribution of long-lasting insecticide-treated nets (LLINs) and indoor residual spraying (IRS) [1]. The country has also made progress in training community health workers on the use of RDTs and ACT [4]. During this time, approximately 24 million LLINs (PermaNet®,Vestergaard Frandsen, and Olyset®,Sumitomo Chemical) were distributed and six million houses were covered with IRS using various chemicals (pyrethroids: lambda cyhalothrin, deltamethrin, alpha-cypermethrin; organochlorine: dichlorodiphenyltrichloroethane (DDT); carbamates: bendiocarb and organophosphates: pirimiphos-methyl) [1,4,5]. The National Malaria Control Centre (NMCC) facilitated the implementation of these control strategies with awareness campaigns and provided information and education communication (IEC) using behaviour change communication (BCC) techniques at the community level.

Zambia has a comprehensive disease surveillance system through the national District Health Information System (DHIS) which includes all public, faith-based and private hospitals as well as rural health centres within an integrated reporting system [4,5]. Of the 14 health facilities in Nchelenge District, 11 report to the surveillance system. The Nchelenge District Health Office (DHO) is responsible for planning, coordinating, managing, implementing, and monitoring health programmes in the District [4].

After six years of implementing malaria control interventions, reductions in malaria infection, illness, severe disease, and death have been reported across much of the country [3,6-9]. However, in some parts of the country there has been limited reduction or even resurgence of malaria, raising concerns about whether recent gains can be sustained and extended [1]. This study reports on the persistent high burden of malaria following the scale-up of malaria control interventions and the presence of insecticide resistance in Nchelenge District, Luapula Province in northern Zambia [3].

## Methods

**Figure 1:**
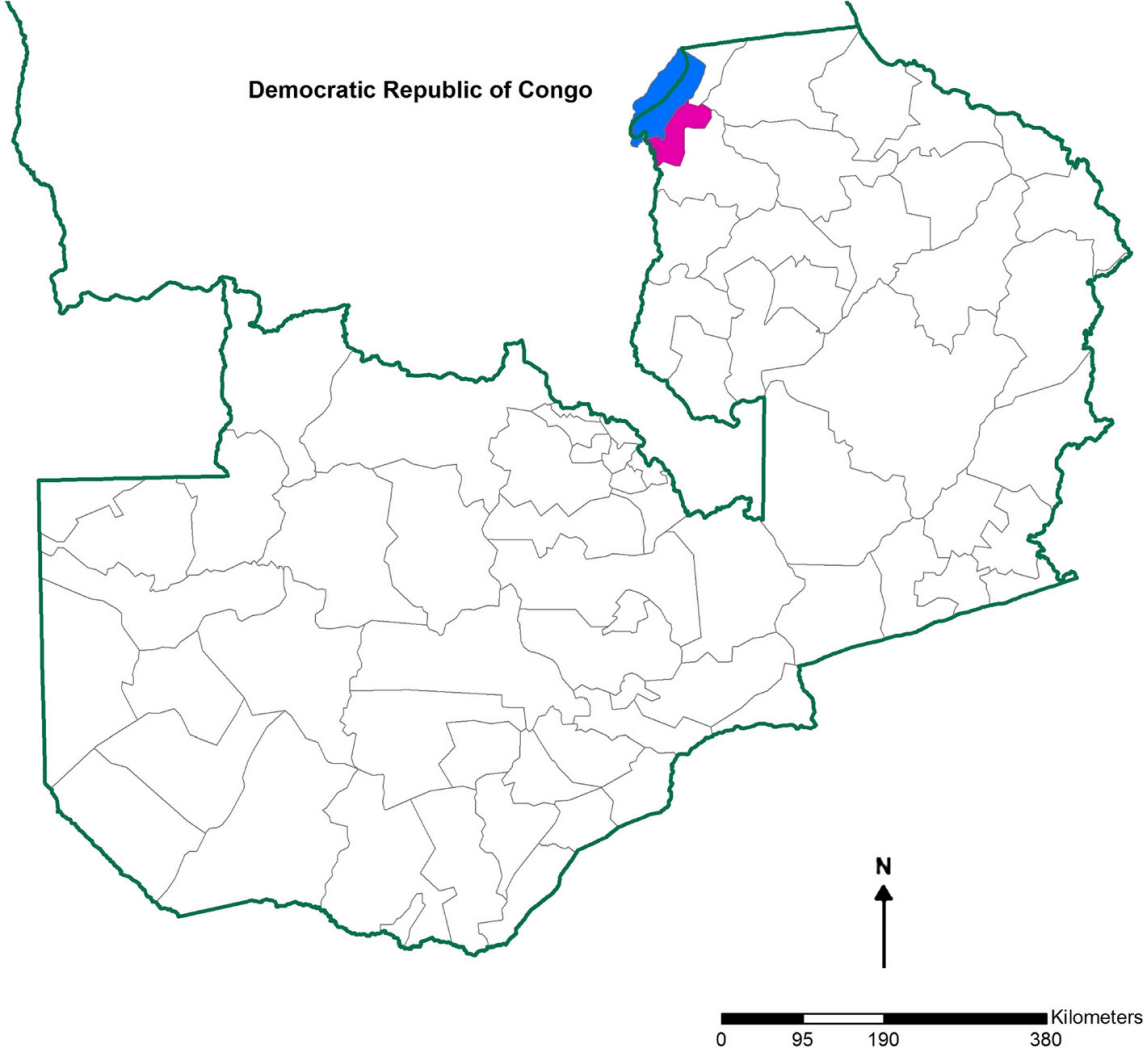
**Location of Nchelenge District.** (Green line indicates an international border. Black line indicates district boundary within Zambia. Blue color indicates area covered by Lake Mweru and pink indicate Nchelenge district in Zambia).

Nchelenge District is in the northwest of Luapula Province in the marshlands of the Luapula River and bordering Lake Mweru, sharing an international border with the Democratic Republic of Congo (Figure 1). Nchelenge has a tropical climate with three seasons: a cool, dry winter (May-August), a hot, dry season (September-October), and a hot, rainy season (November-April) [10]. The population Census in 2010 recorded 147,927 people: 72,797 males and 75,130 females, living in 31,724 houses [11]. Fishing and agriculture are common means of livelihood. Some people engage in fishing, leading nomadic lifestyles and move to agricultural regions when fishing is not permitted.

DHIS data were collected at 11 health facilities in paper form and were sent to the DHO for electronic entry and validation. Information on cases of malaria, malaria deaths, use of malaria diagnostics, and malaria control interventions were obtained from the Nchelenge DHO and consisted of routine surveillance data. The DHO collate the number of malaria cases (malaria is considered to be cases with fever who require anti-malarial treatment [12]) diagnosed by direct microscopy, RDT or based on clinical symptoms [13] reported by health facilities. Yearly aggregated IRS coverage of targeted areas was captured using daily spray forms that were consolidated at the DHO. Data were also available on the number of LLINs distributed annually through all distribution channels, including antenatal and under-fives’ clinics and mass vaccination campaigns. Coverage rate of LLINs (defined as universal access and use of LLINs [14]) was calculated per 1,000 population assuming an average life span of three years [15-17].

Trends in the prevalence of malaria, severe malaria (a set of clinical and laboratory parameters associated with an increased risk of death with the presence of *Plasmodium falciparum* parasitaemia) [18] and malaria-attributable deaths (malaria as the cause of death confirmed by laboratory diagnosis in the hospital) from 2006 to 2012 were assessed for Nchelenge District. Malaria cases were reported annually from 2006 to 2007 and monthly from 2008 to 2012.

Demographic data from the 2000 and 2010 censuses were obtained from the Zambian Bureau of Statistics [11,19] and annual demographic data were projected for 2006 to 2009 and for 2011 to 2012 using an exponential population growth model. The number of houses was projected assuming linear growth. These estimates served as denominators. Descriptive analyses were performed regarding trends in the number of malaria cases, methods of diagnosis, malaria positivity rate among pregnant women, and interventions coverage from 2006 to 2012.

Entomological data were obtained from the Tropical Diseases Research Centre (TDRC) and Luapula Health Office. Entomological data collections were conducted in 2011 and 2012 by the TDRC using pyrethrum spray-catches, mouth-aspirated hand catches and Centers for Disease Control and Prevention (CDC) light-trap methods to determine vector species and indoor densities. Malaria vectors were identified morphologically as *Anopheles gambiae s.l.* and *Anopheles funestus s.l.* using standard keys [20,21]. Insecticide resistance profiles of malaria vectors were determined for 4% DDT and 0.05% deltamethrin using the standard World Health Organization (WHO) tube assay protocol [22].

## Results

**Figure 2:**
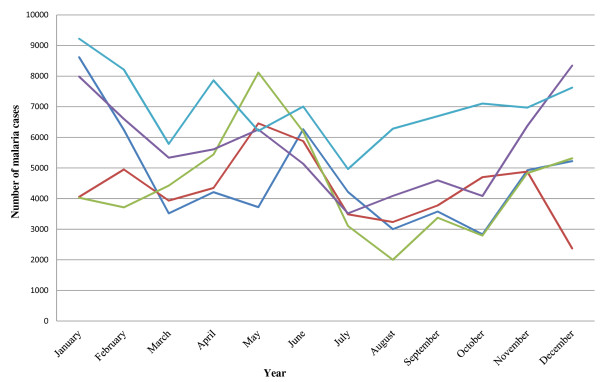
Seasonal distribution of malaria cases in Nchelenge District (Deep blue, red, green, violet and light green line indicate total cases in 2008, 2009, 2010, 2011 and 2012 respectively).

Reported malaria prevalence increased from 38% in 2006 to 53% in 2012 (Table 1). The number of reported malaria cases per year was similar from 2006 to 2010 but increased in 2011 and 2012. Increasing numbers of cases of severe malaria were reported until 2010, with the highest number of deaths (n = 210) reported in 2008 and 2012. Intense seasonal malaria transmission was observed with seasonal declines in the number of cases between April and August, although malaria transmission continued throughout the year (Figure 2).

**Table 1:** Reported malaria burden, diagnostic tests and interventions in Nchelenge district, 2006 to 2012

**Year**	**2006**	**2007**	**2008**	**2009**	**2010**	**2011**	**2012**
Total population	134,363	139,025	143,847	144,957	147,927	152,216	157,118
Number of households	28,782	29,518	30,253	30,989	31,724	32,460	32,500
Number of malaria cases	51,567	46,737	56,355	52,073	53,328	67,923	83,951
Malaria prevalence (%)	38	34	39	36	36	45	53
% malaria cases confirmed by microscopy	5	6	2	2	7	4	3
% malaria cases confirmed by RDT	0	0	18	21	28	68	61
% clinically diagnosed malaria cases	95	94	80	77	65	28	35
Malaria prevalence among pregnant women (%)	**-**	**-**	24	46	33	65	55
LLIN coverage per person	0.07	0.57	1.06	1.55	1.27	1.73	1.24
IRS coverage (% of houses)	0	0	71	89	73	95	19
% of malaria cases that were classified as severe	**-**	**-**	3	4	4	4	2
% of malaria cases resulting in death	0.12	0.18	0.25	0.20	0.16	0.23	0.25

There was an increase in the reported use of microscopy and RDTs to confirm diagnoses, from 5% of cases confirmed by microscopy (prior to the introduction of RDT) in 2006 to 65% of cases confirmed in 2012, with 61% confirmed by RDT. Importantly, the proportion of cases clinically diagnosed declined from 95% in 2006 to 35% in 2012 (Table 1). There was no clear temporal trend for malaria in pregnant women.

Coverage with IRS and LLINs increased from 2006 to 2012. IRS was conducted in 109,095 houses between 2006 and 2012, resulting in coverage of more than 80% of eligible targeted structures between 2006 and 2011. Over the same period, 429,753 LLINs were distributed, resulting in an estimated one LLIN for every two persons in 2007 and higher coverage (1.24 LLIN per person) by 2012 (Table 1). A total of 55,000 nets were distributed in 2007 through the mass distribution programme. Additional nets were distributed in 2011 to replenish those distributed in 2007. The remaining nets were distributed through the malaria in pregnancy programme at health facilities.

In 2011, malaria vector data indicated a preponderance of *An. funestus* with 83% *An. funestus* (N = 185) and 17% (N = 37) *An. gambiae* collected by spray-catches. Using the aspiration method, 280 mosquitoes were collected, of which 96% (N = 260) were *An. funestus* and 4% (N = 20) *An. gambiae*. In 2011, insecticide resistance was detected in *An. funestus* to DDT and deltamethrin. The *An. gambiae* collected were resistant to DDT and deltamethrin. In Zambia, evidence of emerging resistance to pyrethroids and DDT in *An. gambiae s.s.* has been reported since 2009 [3,23].

## Discussion

Despite progress in scaling-up malaria control interventions, a high burden of malaria remains in Nchelenge District in northern Zambia. Similar findings were recognized during the malaria programme review conducted in 2010 by an independent team of experts and concern was expressed about malaria control in Luapula Province, including Nchelenge District [4]. This study shows that the trend has continued and may have worsened. Malaria indicator surveys, which showed a decline in malaria transmission and parasitaemia throughout much of Zambia, identified several transmission foci including Luapula Province [24]. Malaria cases declined in Rwanda following the scale-up of malaria control interventions from 2000 to 2010 [25]. However, gains in malaria control were not sustained in western Kenya [26] nor achieved in Burkina Faso [27].

The recent increase in the reported number of malaria cases could be attributed to increasing insecticide resistance, population movement across borders or internally from endemic areas, increasing outdoor transmission or increased use of RDTs for parasitological confirmation in this highly endemic setting [3,23,28]. The very recent use of RDTs (in 2011 and 2012) coincides with the increased reports of malaria cases during the hot rainy season (Figure 2) so increases may reflect improved diagnostic capabilities to identify less severe cases. Better understanding of the sources of increased reporting and whether continued effectiveness of vector control strategies, transmission ecology, the time and place of infection, and the vectorial capacity in relation to control measures are essential. Routinely collected data demonstrate that Nchelenge District has a high prevalence of malaria but more detailed information and risk maps using geographic information system and remote sensing [29-32] will be required to identify the critical determinants of persistent transmission to target more effective malaria control strategies.

These analyses were based on routinely collected data within the DHIS with the potential for both over and under reporting of malaria cases. Data accuracy and completeness were not systematically assessed. Diagnoses based on clinical signs and symptoms have the potential for misclassification. Both RDT and microscopy have limited sensitivity and specificity, and are particularly likely to misclassify individuals with low levels of parasitaemia.

Although Zambia has made progress with universal coverage of malaria control interventions, there is still need for more targeted interventions and novel strategies in areas with unique characteristics, such as mobile populations in border areas, fishing localities and water bodies with swampy areas, and is consistent with the National Malaria Strategic Plan (NMCC 2011–2015) [33] which recommends selective applications of malaria interventions based on epidemiological trends and status for each region.

## Competing interests

The authors declare that they have no competing interests.

## Authors’ contributions

VMM coordinated collection of data and contributed to critical review and writing of the manuscript. EC, DEN, GEG and WJM contributed to the writing of the manuscript and critically reviewed it. UH conceived the study design, analysed the data and drafted the manuscript. MK, GM, JC, KAC, MM, MC, MM contributed in manuscript writing. All authors read and approved the final manuscript.
